# Retraction: Charcot-Marie-Tooth Disease as a Risk Factor for Periprosthetic Fractures in Tibiotalocalcaneal Fusion With Intramedullary Nailing

**DOI:** 10.7759/cureus.r67

**Published:** 2023-01-20

**Authors:** Ashwin Bhadresha, Baljinder S Dhinsa

**Affiliations:** 1 Trauma and Orthopaedics, William Harvey Hospital, Ashford, GBR

This article has been retracted due to concerns with the accuracy of the data as well as a dispute regarding the correct authorship. The project and data collection were performed by Ashwin Bhadresha (who submitted this article to Cureus) and Karhao Teoh (who is not listed as an author). It was initially submitted to a different journal where it was rejected. Mr. Teoh then expressed concern with the accuracy of the data, but this concern was overlooked by Mr. Bhadresha, who asked a colleague, Dr. Baljinder S. Dhinsa, to review the article prior to submission. The article was then submitted to and published in Cureus without Mr. Teoh or Dr. Dhinsa's consent. The journal was subsequently made aware of this situation by Mr. Teoh, whose allegations were confirmed by Mr. Bhadresha and Dr. Dhinsa. As a result of this series of events, Cureus editorial department has made the decision to retract the article.

